# From Virtual Reconstruction to Additive Manufacturing: Application of Advanced Technologies for the Integration of a 17th-Century Wooden Ciborium

**DOI:** 10.3390/ma16041424

**Published:** 2023-02-08

**Authors:** Daniela Rizzo, Daniela Fico, Francesco Montagna, Raffaele Casciaro, Carola Esposito Corcione

**Affiliations:** 1 Department of Cultural Heritage, University of Salento, via D. Birago 64, 73100 Lecce, Italy; 2 Department of Engineering for Innovation, University of Salento, Edificio P, Campus Ecotekne, s.p. 6 Lecce-Monteroni, 73100 Lecce, Italy

**Keywords:** advanced technologies, cultural heritage, restoration, additive manufacturing, ciborium

## Abstract

3D modelling and 3D printing techniques have become increasingly popular in different fields, including cultural heritage. In this field, there are still many challenges to overcome, such as the difficulty of faithfully reproducing complex geometries or finding materials suitable for restoration, due to the limited scientific studies. This work proposes an example of the application of advanced technologies for the reproduction of four missing columns of a 17th century polychrome wooden ciborium. The difficulties of an automatic scan due to its reflective surface (*water gilding* and *estofado* decorations) were overcome by creating a 2D manual survey and a subsequent manual 3D redrawing. The CAD model was used to print the missing elements with fused filament fabrication (FFF) in polyethylene terephthalate glycol (PETG), using the following printing parameters: nozzle 0.4 mm, infill 20%, extrusion temperature of PLA 200 °C and of PETG 220 °C, plate temperature 50 °C, printing speed 60 mm/s, layer height 0.2 mm. The conservation and restoration of the ciborium is nearing completion. This study highlights the importance of collaboration between different professionals for the correct design of a restoration, as well as the need to promote scientific research into the development of new high-performance 3D printing materials suitable for conservation.

## 1. Introduction

3D printing, also known as rapid prototyping, or additive manufacturing (AM), is an additive technology which enables the creation of a three-dimensional object through working on overlapping layers of materials from 3D models obtained using a specific software (computer-aided design, or CAD) [1]. CAD can digitally create a 2D or 3D object, modify it and share it with other users. The layer-by-layer method enables the creation of complex geometries with optimized, integrated and functional parts with minimal material waste and relatively low speed [2,3]. The additive nature of 3D printing also supports the parallel processing of parts, i.e., multiple parts can be made in a single process in less time than that required for a single creation. This capability allows the production of multiple parts from a single design, multiple revisions of a single part, or multiple parts from different designs. The only limiting factor is the size of the 3D printer’s build area. Thanks to the development of increasingly accurate technologies, cost reductions and research in the field of materials, it is possible to expand its use to numerous sectors [4,5], one of which is cultural heritage [6]. The reproduction of works of art traditionally takes place through a manual process involving the production of rubber molds, which subsequently allow plaster or resin copies to be made by casting, usually on a 1:1 scale. This requires greater care in working on the original works, as well as longer times and higher costs. The use of digital technologies to obtain three-dimensional models and 3D printing makes it possible to work on different scales (e.g., reducing large-scale works to scale), to reproduce details and missing parts, and to obtain an infinite number of reproductions/copies [7,8,9]. Through the use of these technologies, it is also possible to create online museum collections, share CAD models and files for 3D printing, and create new multi-sensory museum routes and educational workshops. Modern methodologies are also used in museum merchandising to produce souvenirs or customized packaging and support structures [6]. There are several 3D printing techniques, and their classification depends on the physical state of the material [10,11,12]. Fused filament fabrication (FFF) or fused deposition modeling (FDM) is the most commonly used additive manufacturing technology for modelling and prototyping. It involves the use of thermoplastic polymer filaments [13,14,15]. There are an increasing number of examples of the application of digital and 3D printing technologies in restoration and in the field of cultural heritage. As reported by Balletti et al. [16], the need to use replicas of works in the field of cultural heritage can be the result of several factors: the restoration and temporary removal of the piece from its original location, its replacement to preserve it from atmospheric conditions that may compromise its integrity, and also the loaning of works for temporary exhibitions. With the aim of preserving the original legibility of the historical site or element to be worked on, the production of 3D-printed copies is increasingly being used. Examples of the application of these techniques are the reconstruction of some missing parts of Antonio Canova’s statue of Hebe in the museum of Bassano del Grappa [17], the reconstruction for the restoration of a Hispano-Roman architectural ornament from the archaeological site of Castulo (located in Spain), or the 3D scanning and 3D printing to support the restoration of the Madonna of Pietranico, a terracotta statue severely damaged in the 2009 Abruzzo earthquake [18]. Additionally, recently exhibited was the 3D printed copy of Michelangelo’s David, made for Dubai World Expo 2020 (https://www.3dnatives.com/en/3d-printed-replica-statue-of-david-dubai-world-expo-051020216/, accessed on 27 December 2022); this work was developed by the Engineering Department of the University of Florence, with the Swedish industrial group Hexagon; it required specialized additive technologies and accomplished the most reliable copy of Michelangelo’s David currently in existence [19]. Additionally, the digital construction site for the reconstruction of Notre Dame de Paris after the fire in 2019 is using 3D technologies [20]. The commercial materials most commonly used with FFF printers are thermoplastic polymers, such as polycarbonate (PC), acrylonitrile butadiene styrene (ABS), polylactic acid (PLA), polyether ether ketone (PEEK) and polyethylene terephthalate glycol (PETG) [21], thanks to their low melting temperature, which allows them to be used in the FFF printing technique (which has a maximum operating temperature of 300 °C). They have good processability, versatility, and adaptability to the printing process, there are a variety of shapes and colours available on the market, and they impart sufficient strength to the final 3D objects [9,22,23]. However, the choice of material to be used in the printing process depends on the object to be printed, what its use will be and its morphological/aesthetic characteristics [24]. Usually, the most commonly used material for 3D printing is polylactic acid (PLA), a biobased, biodegradable and biocompatible thermoplastic polymer that is widely used in the food, medical, cosmetic, agro-industrial, textile, and art sectors, etc. [14,25,26,27,28,29]. Derived from renewable resources such as maize, it has a production capacity of over 140,000 tonnes/year and is hydrolytically degradable [30,31]. Its main characteristics are: extrusion temperature ranging from 160 °C to 230 °C, tensile strength between 35 MPa and 65 MPa, flexural strength of about 97 MPa and a Young’s modulus of 2.3 GPa [9,21]. However, due to certain disadvantages of PLA, such as its high sensitivity to temperature, easy degradability (3–6 months depending on the environmental conditions, size and filling of the object) and hygroscopicity [21,30], a thermoplastic polymer derived from the polyethylene terephthalate family such as polyethylene terephthalate glycol (PETG) is often used. It is well known that exposure to specific conditions of temperature, humidity, ultraviolet radiation and chemicals causes ageing of polymers, resulting in chemical degradation and/or photodegradation [4,9,32,33]. The scientific literature includes many studies on the ageing of PLA and PETG under different atmospheric and chemical conditions [9,33,34,35], whereas studies of accelerated ageing and degradation of 3D printed parts in the same polymeric materials are more limited [33,35]. Furthermore, it must also be considered that printed samples do not always behave like the original raw materials, but more like new systems, with porosity and anisotropic properties [32,35]. For example, Moraczewski et al. (2019) [34] analyzed the effect of accelerated ageing of PLA blends (720, 1440 or 2160 h, 45 °C, 70% relative humidity, in the presence of continuous UV radiation and comparing the results obtained with a commercially available anti-ageing compound), and showed a significant reduction in mechanical properties (e.g., a worse decrease of 17 MPa in tensile strength compared to the unaged sample) [34]. Cuiffo et al. (2017) [35] in their work highlight that the 3D printing process reorganizes the molecular polymer chains of PLA, causing an increase in the water sensitivity of the material [35]. Moreno Nieto et al. (2021) [36] studied the durability and degradation in different aquatic environments of PLA and PETG-printed samples (with the FFF technique). Specifically, the samples were immersed in a solution of distilled water and in two saturated solutions of sea salt and sugar, and the degradation and water absorption behavior of each sample was monitored periodically in terms of size and weight. Percentage water absorption rates of 2.5% for PLA and 0.3% for PETG were recorded, showing that PLA degrades much more easily than PETG, and that PETG has greater stability [36]. PETG is a thermoplastic polymer consisting of polyethylene terephthalate (PET) and ethylene glycol, known for its ductility and mechanical strength [37,38,39,40]. The main characteristics of PETG are an extrusion temperature ranging from 220 °C to 250 °C, a tensile strength of 49 MPa and a flexural strength of approximately 70 MPa [9,21]. It is a particularly strong polymer with better mechanical properties than PLA; these characteristics drove the choice of this material for this study [9,21,41]. PETG is not always preferred over PLA or other polymers in restorations, partly due to cost implications that often influence the initial choice of material to be used, and studies on the variation of mechanical properties as a function of the parameters of the 3D printing process are still generally limited [42,43].

This research represents a proof of concept aimed at demonstrating the suitability of FFF for the reproduction and restoration of ancient artifacts. The present work focuses on the reproduction of the four missing columns of a 17th-century polychrome wooden ciborium belonging to a private collection. The research is finalized to an integrative restoration, not just to a conservation work. However, the aim is also the preservation and maintenance of the object without altering its original parts. It is developed using FFF printing technologies and a profitable collaboration between professional figures belonging to different disciplinary fields. This aspect allows us to demonstrate several advantages, such as the possibility of integrating missing parts of the object (improving its legibility and comprehension), thereby reducing the cost of raw materials and the time required to realize the artistic element compared to the use of traditional manual reproduction techniques.

## 2. Materials and Methods

### 2.1. Case Study: Ciborium

**Figure 1:**
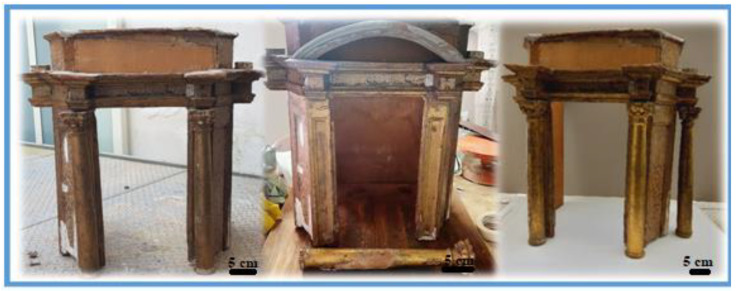
The 17th-century *ciborium* studied and its state of conservation. Scale bar equal to 5 cm.

The ciborium is an architectural element in the form of a canopy above the high altar in medieval and Renaissance Christian churches, usually made of marble or wood. Generally, the ciborium is supported by four vertical supports or columns connected by arches, supporting a flat vault or dome, intended to hold the pyx containing the consecrated hosts. The studied ciborium (Figure 1), according to the style and decorative patterns, can probably be ascribed to the 17th century.

The art object belongs to a private collector who bought it from a Neapolitan junk dealer, which is why it is not catalogued. It is made of wood, and due to its state of conservation (consisting of the absence of four of the six original columns and in some areas of pictorial gaps and patinas) it requires restoration. The ciborium is decorated with *water gilding* and *estofado* decorations; the latter term derives from the Catalan word “*estofar*” which meant, for the gilders of the time, scraping the layer of painting spread on the gilding with an awl, a technique widely used in the decoration of polychrome wooden sculpture. This rich technique, whose origin can be traced back to Siena in the fourteenth century, was largely used in Spain, which is why it is mainly known through a Spanish name. It probably arrived in Naples in the mid-fifteenth century and was widely used in the following two hundred years. The complex workmanship consisted of covering the carved surface with four layers of materials: a first layer of gesso and glue, then a layer of bole (normally red or Armenian bole), over which a very thin layer of gold leaf was spread, followed by a layer of paint. The latter was then partially scratched in such a way as to trace a design, bringing out the gold underneath. 

### 2.2. Experimental Procedure

**Figure 2:**
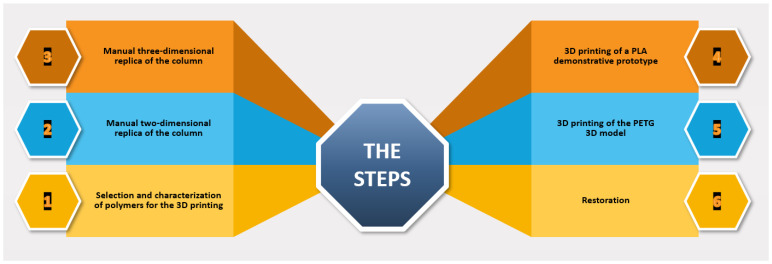
Summary diagram of the different steps of the complete experimental work used in this study.

The multidisciplinary experimental work performed in this paper consisted in the following steps (Figure 2):

- Selection and characterization of polymers for the 3D printing;
- Manual two-dimensional replica of the column;
- Manual three-dimensional replica of the column;
- 3D printing of a PLA demonstrative prototype;
- 3D printing of the PETG 3D model; and
- Restoration.

#### 2.2.1. Selection and Characterization of Polymers for the 3D Printing

In order to select the material with the appropriate aesthetic and durability features required by the restorer for the reproduction of the missing columns of the ciborium, a preliminary characterization of the polymeric materials to be used in fused filament fabrication (FFF) printing was carried out. The specific requirements of the material used for this kind of application are easy processability and printability, good hardness, impact, ductility and chemical resistance, and thermal stability. The results are reported in the Supplementary Materials Section S1. Specifically, in accordance with the previously reported scientific literature [21,41], two materials were selected (polylactic acid-PLA and polyethylene terephthalate glycol-PETG, respectively). Their thermal and mechanical properties were investigated for the final choice of material and are again reported in Supplementary Materials Section. Finally, two prototypes of the column were printed. A polylactic acid (PLA) filament purchased from the company Fabbrix^®^ (Ruvo di Puglia, Bari, Italy) was used to print the first demonstration prototype of one of the ciborium columns. According to the producer’s technical data sheet, it has a diameter of 1.75 ± 0.05 mm, a density of 1.25 g cm^−3^ and a melt flow index (MFI) of 7–9 g/10 min at a temperature of 190 °C. PLA filament was selected for its biodegradability, low cost and easy processability, even if it is not suitable for the realization of the final 3D model. PLA, in fact, has low mechanical properties and durability, because of its high hygroscopicity. The final model was, in fact, realized using polyethylene terephthalate glycol (PETG) filament from the company PrimaSELECT (Malmo, Sweden). According to the producer’s technical data sheet, it has a diameter of 1.75 ± 0.05 mm, a density of 1.27 g cm^−3^ and a melt flow index (MFI) of 12.1 g/10 min at a temperature of 225 °C. PETG filament has superior thermo-mechanical characteristics (such as hardness, impact, ductility chemical resistance and thermal stability) compared to PLA. It has a lower processability and printability compared to PLA. However, it can still be considered suitable for the selected low cost FFF printer.

#### 2.2.2. Manual Two-Dimensional and Manual Three-Dimensional Replicas of the Column

An accurate two-dimensional reproduction of the column was carried out manually. The poor state of preservation of the ciborium and the extremely reflective surfaces precluded a virtual scan. For the reproduction, the measurements were taken with the use of a caliber and the photographs from different angles of the column were used, in order to accurately draw the decorative parts, such as the capital. Afterwards, the CAD model of the column was obtained using Rhinoceros software (Robert McNeel & Associates, Seattle, WA, USA) and subsequently modified with Fusion 360 software (Autodesk, San Rafael, CA, USA). Cura software (Ultimaker B.V., Utrecht, The Netherlands) was used to convert the CAD file to an .STL file and for the slicing process. 

#### 2.2.3. D Printing

The demonstrative prototype of the ciborium column in PLA and the functional model in PETG were printed using a 3DPRN LAB printer (3DPRN company, Castiglione M.R., Italy) [14,44] and the following process parameters: nozzle 0.4 mm, infill 20%, extrusion temperature of PLA 200 °C and of PETG 220 °C, plate temperature 50 °C, printing speed 60 mm/s, printing cooling (enable for PLA and not enable for PETG), layer height 0.2 mm. The part was oriented on the printer platen in such a way as to minimize the supports for the cantilevered parts of the object. 

#### 2.2.4. Restoration Materials

The following materials were used for restoration work on the original and copied columns: Bologna gypsum (CTS srl, Bari, Italy), rabbit glue (CTS srl, Bari, Italy), putty (modostuc Gimod srl, Pavia, Italy), yellow ochre bolus (CTS srl, Bari, Italy), gelatin sheets (Dolciaria Pezzella srl, Naples, Italy), Paraloid B72 (Restauro Tecnica Srls, Vicenza, Italy), nitro thinner (CTS srl, Bari, Italy), 22K gold leaf, 917/1000, dim. 8 × 8 cm (Aurum SAS gilding products, Bologna, Italy).

## 3. Results

### 3.1. Manual Two-Dimensional and Manual Three-Dimensional Replicas of the Column

**Figure 3:**
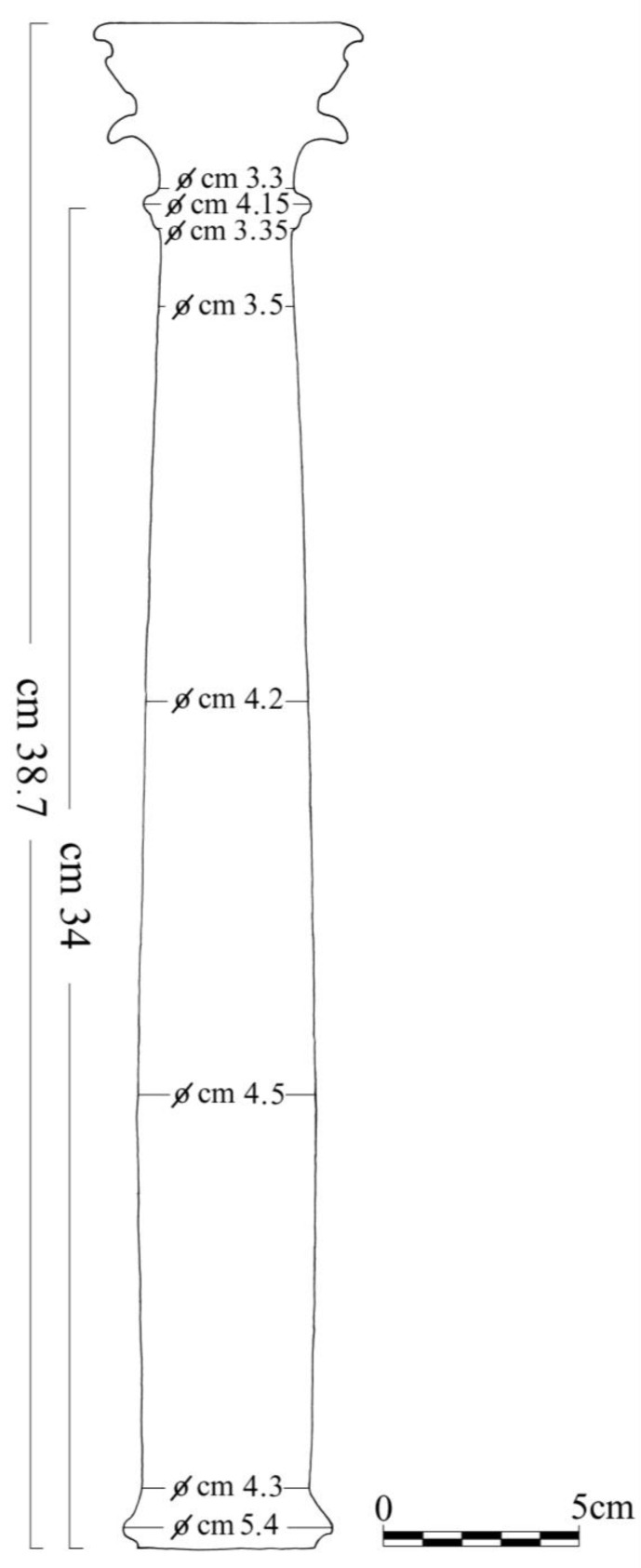
Manual reproduction of column with component measurements (Scale bar equal to 5 cm).

A photogrammetric mapping of the ciborium column was performed using a DSC-W570 camera (Sony Europe B.V., United Kingdom). The two-dimensional manual drawing of the column was made with the help of photographs of different angles of the object and the use of a caliber (Figure 3).

**Figure 4:**
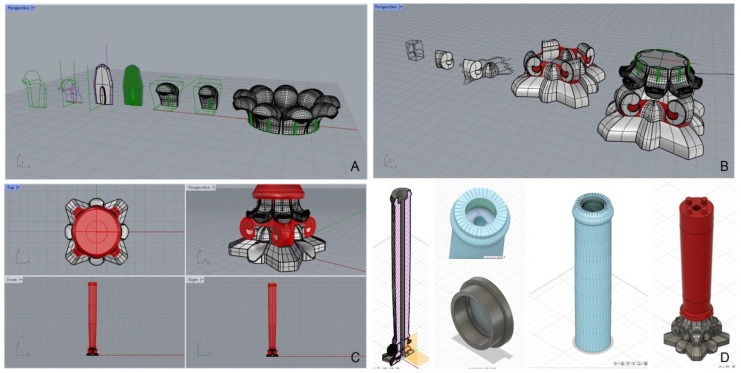
Steps of three-dimensional modeling of the column: modeling of the volutes (**A**) and the assembled capital (**B**) using Rhinoceros software; 3D model of the upper section of the column, front of the capital and whole column using Rhinoceros software (**C**); transversal section of the column (left) and modeling (right) of structural components using Fusion 360 software (**D**).

Subsequently, these data were imported into the Rhinoceros 3D modeling software to create 3D model of column (Figure 4A–C). Then, the optimal 3D printing procedure was identified after the creation of the CAD drawing that faithfully reproduced the artefact, including the decorations of the capital. The original column had dimensions larger than the working volume of the FFF printer (200 mm × 200 mm × 200 mm), so it was necessary to divide the 3D model in half height (base and capital) into two parts and create two separate CAD files. This operation was carried out using Fusion 360 software (Figure 4D). The model was first sectioned parallel to the column section at mid-height, and then joints were added so that the two parts could be brought together after printing. In addition, a center hole was added so that a threaded aluminum bar could be inserted after assembly. This bar served as reinforcement to increase the mechanical strength, to support the load of the ciborium structure (lintel), and to join the two parts by means of a clamping nut. A cover, also designed using Fusion 360 software and fabricated by FFF printing, was finally added to hide the hole (Figure 4D). Finally, the slicing operation was performed by transforming the CAD file into STL format and then formed into GCode, using Ultimaker Cura software. The printing strategy used made it easier to place the object on the printer plate, minimizing supports, with less material waste. It also made it possible to have layers perpendicular to the height of the column, allowing, during the final restoration phase, a good grip of the filler applied by the restorer, to recreate the original texture.

### 3.2. D Printing of a PLA Demonstrative Prototype

**Figure 5:**
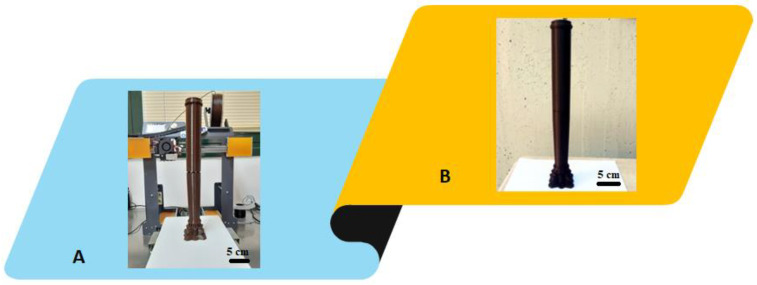
Prototypes printed with PLA (**A**) and final model of column in PETG (**B**). Scale bar equal to 5 cm.

A demonstrative prototype of the 3D model obtained in the previous step, was firstly printed by using PLA and 3DPRN LAB printer (Figure 5). This step was necessary only to verify the suitability of the proposed methodology to obtain a 3D reproduction similar to the original. A surface-finishing operation followed, through the removal of the supports present exclusively in the decorated capital. Through visual observation of the PLA prototype, good adhesion of the different print layers was revealed, and the presence of any defects/holes was identified; these were remodeled to create the final PETG model.

### 3.3. D Printing of the PETG 3D Model

**Figure 6:**
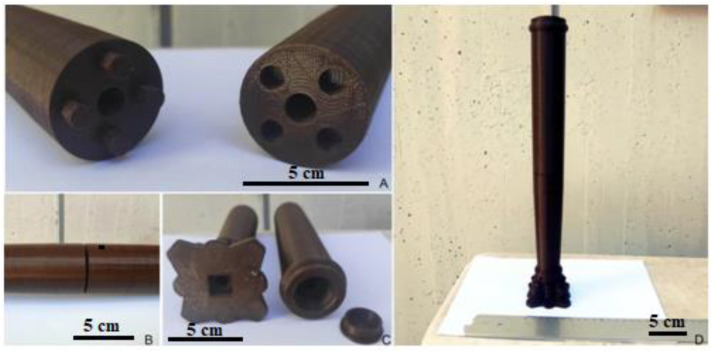
3D model of column in PETG: grooves and hole for the threaded aluminium bar at half height of the shaft (**A**), detail of the two parts of the column joined together thanks to the grooves (**B**), holes for the threaded aluminium bar and cap (**C**) and assembled column (**D**). Scale bar equal to 5 cm.

Based on the results obtained from the thermal and mechanical characterization of the two materials (reported in the Supplementary Materials, Section S1), and the refinement of the model from the analysis of the PLA prototype, PETG columns were finally printed (Figure 6). The printing took about 5 h and 30 min for the lower part (base) and about 7 h and 20 min for the upper part (capital) for each column. In contrast, the printing of the lid took about 20 min.

### 3.4. Restoration

**Figure 7:**
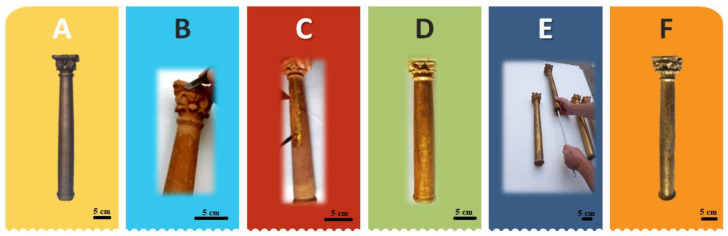
Main phases of column restoration: copy of column (**A**), modelling the capital with stucco using a scalpel (**B**), application of gold leaf on bolus (**C**), copy of column with gouache gilding (**D**), aluminium bar insertion operation (**E**), original column with retouching by striping or hatching (**F**). Scale bar equal to 5 cm.

As previously mentioned, the uncatalogued object, which belongs to a private owner, was lacking four of the original six columns, which is why an integrative restoration was chosen through the reconstruction of the missing columns by means of 3D printing in PETG. Different operations were carried out on the two original columns and the copies in order to ensure compliance with the so-called five fundamental principles of restoration: recognizability, reversibility, compatibility, minimum intervention and interdisciplinarity. In fact, every restoration must be recognisable; therefore, any added part must be distinguishable from the original, without disturbing the overall look of the work. The phases of the conservation and aesthetic restoration of the original columns have included the consolidation of the wood with Paraloid B72, filling with Bologna gypsum and rabbit glue in lacking areas, and painting interventions aimed at formal chromatic balance. Pictorial retouching was carried out in “rigatino”, using the concept of chromatic selection, i.e., with fine hatching, which is recognisable compared to the original when observing the object from close up; from a distance, it allows a substantially unified reading of the decoration. On the PETG-printed copies of the column, a mimetic intervention was carried out, which involved preparing the background with Bologna plaster and rabbit glue, modelling with a scalpel, drafting the red bolus and the application of gold leaf with fish glue and finally burnishing to ensure its lustre over time (Figure 7).

By restoration, we mean any type of intervention aimed at restoring a product of human activity, which has the purpose of recovering the historical and artistic significance of the work or is aimed at restoring the functionality of the artefact, or both, guaranteeing the physical integrity and legibility of the work and respecting the fundamental principles of restoration.Thanks to the actions undertaken, it was possible to respect this statement but also to meet the aesthetic taste of the object’s owner. It is possible, in fact, to distinguish the original columns from the copies by the cracking, for example, which is a type of degradation that affects the work from both a conservation and aesthetic point of view. The movement of the wood contrasts with the rigidity of the preparation and causes this phenomenon. Another difference that allows us to distinguish between the original and the restored elements is the natural ageing of the first, compared with the lustre of the newly applied gold on the reproduction. The structure is also different, because the added columns are reinforced with aluminium bars.

## 4. Conclusions

The worlds of cultural heritage, technology, and materials are increasingly intertwined. This experimental work demonstrates the potential suitability of 3D printing techniques for the conservation of and reproduction of cultural and artistic heritage objects, offering users the opportunity to physically see works of art in their former glory and not just imagine them. This work concentrates on the application of 3D advanced technologies for the reproduction and integration of missing elements of the historical art object. A 17th-century wooden ciborium was selected as a case study. The following steps were necessary to reach the aim:

- Creation of a manual 2D survey, followed by a manual 3D redesign with Rhinoceros software, which was used to print the missing elements by fused filament fabrication (FFF).
- Preliminary experimental characterization of two of the most used 3D printing materials in order to select polyethylene terephthalate glycol (PETG) for the construction of the final 3D model.
- Restoration of the 3D printed column, applying preliminary plastering and proceeding with gouache gilding and strip or hatching retouching. A metal bar was inserted inside the finished columns to make them more resistant to stress.

In conclusion, this study, through experimental reintegration of missing parts, showed how the combination of digital techniques and traditional restoration techniques, as well as interdisciplinary skills, allows the restorer to continue a project that he would otherwise have abandoned. The main advantage of additive technologies is that it is possible to produce parts with any geometry at any time in the case of small batch requests, one-off and on-demand parts, and finished, functional parts of an object. The ability to print spare parts or components of any kind within a few hours and with reduced costs can help to avoid losses due to downtime and significantly reduce maintenance costs. In short, all this guarantees the possibility of improving the study and enjoyment of heritage. 

## Data Availability

Not applicable.

## Supplementary Materials

The following supporting information can be downloaded at: https://www.mdpi.com/article/10.3390/ma16041424/s1.

## References

[1] Vyavahare S., Teraiya S., Panghal D., Kumar S. (2020). Fused Deposition Modelling: A Review. Rapid Prototyp. J..

[2] Ahmad M.N., Ishak M.R., Mohammad Taha M., Mustapha F., Leman Z., Anak Lukista D.D., Irianto, Ghazali I. (2022). Application of Taguchi Method to Optimize the Parameter of Fused Deposition Modeling (FDM) Using Oil Palm Fiber Reinforced Thermoplastic Composites. Polymers.

[3] Figueiredo L.F., Vieira F.S., Jamieson O.D., Reeder J., Mc Lean T., Olsen J., Crapnell R.D., Whittingham M.J., Banks C.E., Law R. (2022). Influence of Design and Material Characteristics on 3D Printed Flow-Cells for Heat Transfer-Based Analytical Devices. Microchim. Acta.

[4] Ferrari F., Striani R., Fico D., Alam M.M., Greco A., Esposito Corcione C. (2022). An Overview on Wood Waste Valorization as Biopolymers and Biocomposites: Definition, Classification, Production, Properties and Applications. Polymers.

[5] Esposito Corcione C., Palumbo E., Masciullo A., Montagna F., Torricelli M.C. (2018). Fused Deposition Modeling (FDM): An Innovative Technique Aimed at Reusing Lecce Stone Waste for Industrial Design and Building Applications. Constr. Build. Mater..

[6] Balletti C., Ballarin M. (2019). An Application of Integrated 3D Technologies for Replicas in Cultural Heritage. ISPRS Int. J. Geo-Inform..

[7] Anchez-belenguer C.S., Val D., Anchez-l M.S., Carmen D., Carlos S., Carmen D. (2015). Automatic Production of Tailored Packaging for Fragile Archaeological Artifacts. J. Comput. Cult. Herit..

[8] Higueras M., Calero A.I., Jos F. (2021). Digital 3D Modeling Using Photogrammetry and 3D Printing Applied to the Restoration of a Hispano-Roman Architectural Ornament. Digit. Appl. Archaeol. Cult. Herit..

[9] Fico D., Rizzo D., Casciaro R., Esposito Corcione C. (2022). A Review of Polymer-Based Materials for Fused Filament Fabrication (FFF): Focus on Sustainability and Recycled Materials. Polymers.

[10] Klippstein H., Diaz De Cerio Sanchez A., Hassanin H., Zweiri Y., Seneviratne L. (2018). Fused Deposition Modeling for Unmanned Aerial Vehicles (UAVs): A Review. Adv. Eng. Mater..

[11] Levy G.N., Schindel R., Kruth J.P. (2003). Rapid Manufacturing and Rapid Tooling with Layer Manufacturing (LM) Technologies, State of the Art and Future Perspectives. CIRP Ann.-Manuf. Technol..

[12] Grzegorz Gaweł T. (2020). Review of Additive Manufacturing Methods. Solid State Phenom..

[13] Medellin-Castillo H.I., Zaragoza-Siqueiros J. (2019). Design and Manufacturing Strategies for Fused Deposition Modelling in Additive Manufacturing: A Review. Chin. J. Mech. Eng. (Engl. Ed.).

[14] Fico D., Rizzo D., De Carolis V., Montagna F., Esposito Corcione C. (2022). Sustainable Materials and Technologies Sustainable Polymer Composites Manufacturing through 3D Printing Technologies by Using Recycled Polymer and Filler. Polymers.

[15] Fico D., Rizzo D., De Carolis V., Montagna F., Palumbo E., Esposito Corcione C. (2022). Development and Characterization of Sustainable PLA/Olive Wood Waste Composites for Rehabilitation Applications Using Fused Filament Fabrication (FFF). J. Build. Eng..

[16] Balletti C., Ballarin M., Guerra F. (2017). 3D Printing: State of the Art and Future Perspectives. J. Cult. Herit..

[17] De Crescenzio F., Fantini M., Persiani F., Virgilli V., Santopuoli N., Seccia L. (2010). Restauro L’Ebe di Canova: Modello Digitale e Sviluppi Applicativi. Archeomatica.

[18] Irene G. (2018). La Stampa 3D Nei Beni Culturali Analisi e Caratterizzazione Di Materiali per La Fabbricazione Digitale Di Beni Culturali.

[19] A 3D Printed Replica of Michelangelo’s Statue of David Presented at the Dubai World Expo. https://www.3dnatives.Com/En/3d-Printed-Replica-Statue-of-David-Dubai-World-Expo-051020216/.

[20] Olden K. (2021). A futuristic simulation of Notre Dame to power its real world resurrection. Domus.

[21] Algarni M., Ghazali S. (2021). Comparative Study of the Sensitivity of Pla, Abs, Peek, and Petg’s Mechanical Properties to Fdm Printing Process Parameters. Crystals.

[22] Cano-Vicent A., Tambuwala M.M., Hassan S.S., Barh D., Aljabali A.A.A., Birkett M., Arjunan A., Serrano-Aroca Á. (2021). Fused Deposition Modelling: Current Status, Methodology, Applications and Future Prospects. Addit. Manuf..

[23] Rett J.P., Traore Y.L., Ho E.A. (2021). Sustainable Materials for Fused Deposition Modeling 3D Printing Applications. Adv. Eng. Mater..

[24] Scopigno R., Cignoni P., Pietroni N., Callieri M., Dellepiane M. (2017). Digital Fabrication Techniques for Cultural Heritage: A Survey. Computer Graphics Forum.

[25] Muhammad A., Rashidi A.R., Roslan A., Idris S.A. (2017). Development of Bio Based Plastic Materials for Packaging from Soybeans Waste. AIP Conf. Proc..

[26] Muñoz V.G., Muneta L.M., Carrasco-Gallego R., Marquez J.D.J., Hidalgo-Carvajal D. (2020). Evaluation of the Circularity of Recycled PLA Filaments for 3D Printers. Appl. Sci..

[27] Esposito Corcione C., Gervaso F., Scalera F., Padmanabhan S.K., Madaghiele M., Montagna F., Sannino A., Licciulli A., Maffezzoli A. (2019). Highly Loaded Hydroxyapatite Microsphere/ PLA Porous Scaffolds Obtained by Fused Deposition Modelling. Ceram. Int..

[28] Esposito Corcione C., Scalera F., Gervaso F., Montagna F., Sannino A., Maffezzoli A. (2018). One-Step Solvent-Free Process for the Fabrication of High Loaded PLA/HA Composite Filament for 3D Printing. J. Therm. Anal. Calorim..

[29] Murariu M., Paint Y., Murariu O., Laoutid F., Dubois P. (2022). Recent Advances in Production of Ecofriendly Polylactide (PLA)–Calcium Sulfate (Anhydrite II) Composites: From the Evidence of Filler Stability to the Effects of PLA Matrix and Filling on Key Properties. Polymers.

[30] Vaidya A.N., Pandey R.A., Mudliar S., Kumar M.S., Chakrabarti T., Devotta S. (2005). Production and Recovery of Lactic Acid for Polylactide-An Overview. Crit. Rev. Environ. Sci. Technol..

[31] Carrasco F., Pagès P., Gámez-Pérez J., Santana O.O., Maspoch M.L. (2010). Processing of Poly(Lactic Acid): Characterization of Chemical Structure, Thermal Stability and Mechanical Properties. Polym. Degrad. Stab..

[32] Amza C.G., Zapciu A., Baciu F., Vasile M.I., Nicoara A.I. (2021). Accelerated Aging Effect on Mechanical Properties of Common 3d-printing Polymers. Polymers.

[33] Głowacki M., Mazurkiewicz A., Słomion M., Skórczewska K. (2022). Resistance of 3D-Printed Components, Test Specimens and Products to Work under Environmental Conditions—Review. Materials.

[34] Moraczewski K., Stepczyńska M., Malinowski R., Karasiewicz T., Jagodziński B., Rytlewski P. (2019). The Effect of Accelerated Aging on Polylactide Containing Plant Extracts. Polymers.

[35] Cuiffo M.A., Snyder J., Elliott A.M., Romero N., Kannan S., Halada G.P. (2017). Impact of the Fused Deposition (FDM) Printing Process on Polylactic Acid (PLA) Chemistry and Structure. Appl. Sci..

[36] Moreno Nieto D., Alonso-García M., Pardo-Vicente M.A., Rodríguez-Parada L. (2021). Product Design by Additive Manufacturing for Water Environments: Study of Degradation and Absorption Behavior of Pla and Petg. Polymers.

[37] Marković M.P., Cingesar I.K., Keran L., Prlić D., Grčić I., Vrsaljko D. (2022). Thermal and Mechanical Characterization of the New Functional Composites Used for 3D Printing of Static Mixers. Materials.

[38] Shi Q., Xiao R., Yang H., Lei D. (2020). Effects of Physical Aging on Thermomechanical Behaviors of Poly(Ethylene Terephthalate)-Glycol (PETG). Polym. Technol. Mater..

[39] Colón Quintana J.L., Slattery L., Pinkham J., Keaton J., Lopez-Anido R.A., Sharp K. (2022). Effects of Fiber Orientation on the Coefficient of Thermal Expansion of Fiber-Filled Polymer Systems in Large Format Polymer Extrusion-Based Additive Manufacturing. Materials.

[40] Ronca A., Abbate V., Redaelli D.F., Storm F.A., Cesaro G., De Capitani C., Sorrentino A., Colombo G., Fraschini P., Ambrosio L. (2022). A Comparative Study for Material Selection in 3D Printing of Scoliosis Back Brace. Materials.

[41] Vidakis N., Petousis M., Velidakis E., Liebscher M., Mechtcherine V., Tzounis L. (2020). On the Strain Rate Sensitivity of Fused Filament Fabrication (Fff) Processed Pla, Abs, Petg, Pa6, and Pp Thermoplastic Polymers. Polymers.

[42] Scianna A., Filippo G. (2019). Di Rapid Prototyping for the Extension of the Accessibility to Cultural Heritage for Blind People. Int. Arch. Photogramm. Remote Sens. Spatial Inf. Sci..

[43] Catic I., Caloska J., Godec D., Kovacic M., Pilipovic A., Skala K. (2017). Fluid-Deposition of Rocks Is Natural Model for Additive Production. Interdiscip. Descr. Complex Syst..

[44] Ferrari F., Esposito Corcione C., Montagna F., Maffezzoli A. (2020). 3D Printing of Polymer Waste for Improving People’s Awareness about Marine Litter. Polymers.

