# Supplementary material for: From Virtual Reconstruction to Additive Manufacturing: Application of Advanced Technologies for the Integration of a 17th-Century Wooden Ciborium
Source: Materials (Basel). 2023 Feb 8;16(4):1424. doi: 10.3390/ma16041424 (PMC9965309; doi:10.3390/ma16041424)
Supplement: Supplementary file 1 [file materials-16-01424-s001.zip › materials-2164299-supplementary.pdf]

## Supplementary materials S1

Experimental techniques

The thermal characterization of the polymeric materials (polylactic acid-PLA and polyethylene terephthalate glycol-PETG) selected for Fused Filament Fabrication (FFF) printing, and the study of the thermal and mechanical properties of the respective printed samples, was carried out using the following analytical methodologies:

- DSC analysis (Mettler Toledo DSC1 StareSystem, Milano, Italy) was performed on PLA and PETG filaments and 3D printed bars, to investigate the thermal features of materials, by measuring glass transition temperature ($T_g$), crystallization temperature ($T_c$) and melting temperature ($T_m$). The analyses were conducted using a temperature range of 25°C to 200°C, at a heating rate of 10°C/min.

- The Netzsch STA 409 simultaneous thermal analyser (Netzsch, Selb, Germany) was used for thermogravimetric characterisation (TGA) of the two filaments. The samples were heated in the range of 20°C to 700°C, heating rate of 10°C/min in air.

- 3D samples used for mechanical tests were printed using the Creality CP-01 printer (Creality, London, UK) and setting the following operating conditions: extrusion temperature 200°C for PLA and 220°C for PETG, printing speed 50 mm/s, infill 20%. The CAD model was created with Fusion 360 software (Autodesk, San Rafael, CA, USA), which was converted to an .STL file using Cura software (Ultimaker B.V., Utrecht, The Netherlands).

- According to the ISO178(2014) standard, flexural tests were performed on all 3D printed bars using a Lloyd LR5K dynamometer (Lloyd Instruments Ltd., Bognor Regis, UK), with a test speed of 2 mm/min and a specimen support spacing of 64 mm. Finally, tensile tests were performed on the 3D printed samples, of dimensions of 60 mm x 13.2 mm x 0.7 mm, using the same dynamometer. Six replicates for each mechanical measurement were performed, in order to obtain statistically relevant results.

Results

A careful study of the thermal properties and stability of the PLA and PETG was carried out, in order to confirm the literature data and to select the most suitable material for 3D printing. DSC analysis was performed to verify the difference in the crystal structure of the two filaments. The glass transition temperature ($T_g$) was calculated by inflection point method, and $T_g$ coincides with the point where the second derivative is zero. As expected, the DSC curves (Figure S1A) of the two polymers are very different, like the thermal properties, crystal structure, molecular weight and mobility of the polymer chains. The glass transition temperature values $T_g$ measured for PLA and PETG are 56.5°C and 71.0°C (Figure S1B), respectively, comparable to those reported in the literature [1–5]. A higher degree of crystallinity and a proximity of the polymer chains to the crystallites corresponds to a higher glass transition temperature, as can be seen for PETG [6,7]; the mechanism of polymer crystallization also depends on the type of fillers, fibers and plasticizers possibly used, as well as, the induced nucleation [7,8], information not known from the manufacturers' data sheets. For example, Cano-Vicent et al. [3] reported a value of $T_g$ approximately 74°C for PETG. Shi et al. [9] reported variations in the $T_g$ value between 70°C and 90°C for PETG, depending on different thermal and degradation conditions, while Quintana et al. [8] reported a value of glass transition of 77.5°C for neat PETG and 75.9°C for PETG with added glass fibres (GF). Marković et al. [10] report a glass transition temperature range for PETG filaments between 73°C and 75°C, for pure PETG or related titanium dioxide ($TiO_2$) and carbon nanotube (CNT)-based composites. Also for PLA, for example Carrasco et al. [11] report in their study variations of $T_g$ from 60°C up to 68°C, depending on the different conditions of extrusion and injection of the polymer, or Esposito Corcione et al. [12] report an increase of $T_g$ until to 66°C

with the addition of inorganic filler (limestone), while Fico et al. [13] indicate a reduction of the $T_g$ until to 51.3°C with the addition of wood particles to matrix. The crystallization of PLA starts at lower temperatures ($T_c$=95.5°C) than PETG ($T_c$=117.6°C), as does the melting process ($T_m$ of PLA equal to 168.1°C, of PETG equal to 253.8°C); also for this reason, in addition to biodegradability, polylactic acid is often preferred for 3D printing. The curves related to the TGA analysis performed on both filaments are shown in Figure S1D. The results indicate a reduction in thermal stability for PLA at temperatures above 300°C, while the data highlight a greater range of thermal stability for PETG, which degrades above 400°C. Table S1 shows additional information obtained from the TGS curves of the filaments.

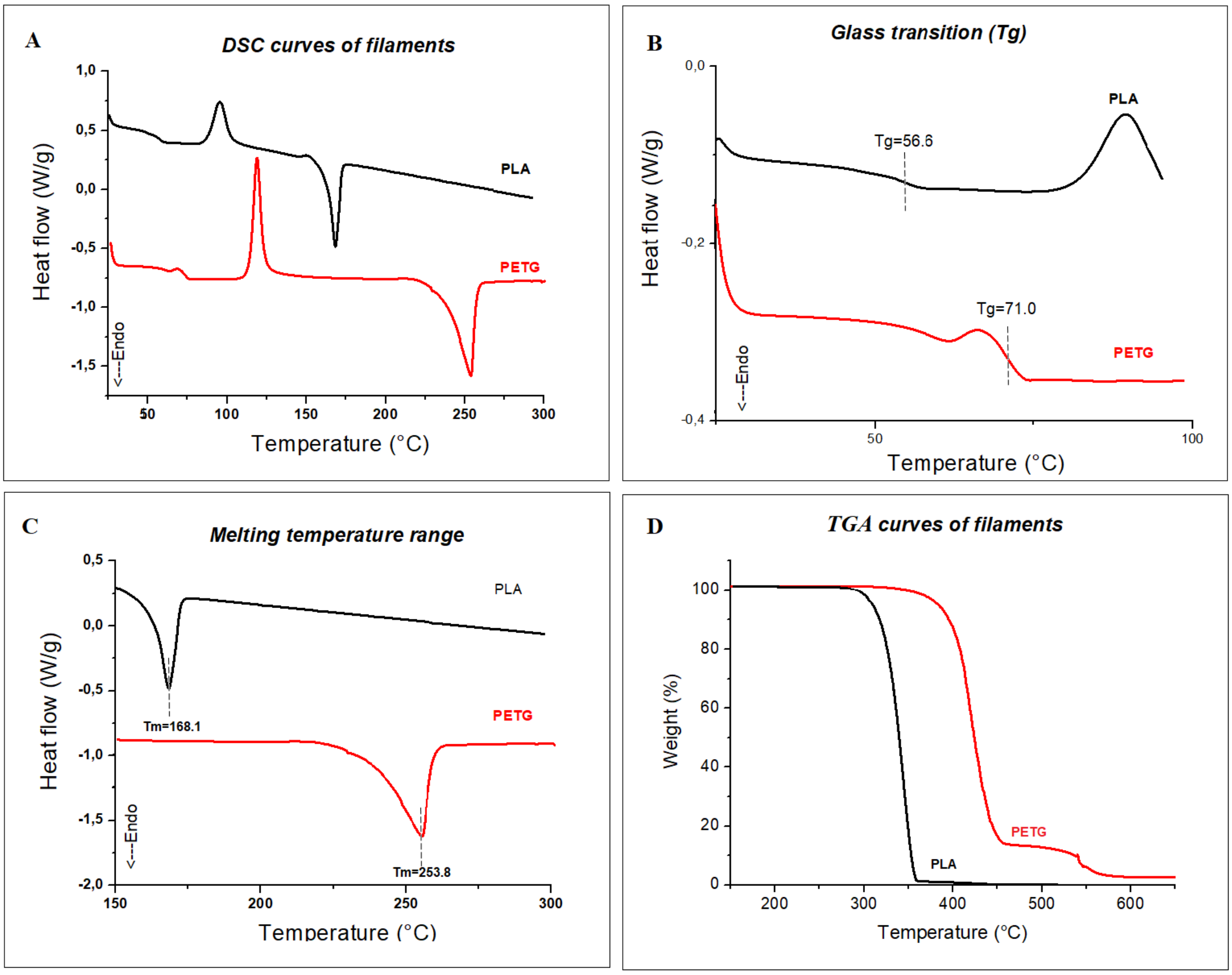


**Figure S1.** DSC analysis of filaments (A), Glass transition (B), Melting temperature range (C) and TGA curves on filaments (D).

**Table S1.** $T_{onset}$ (°C) and Solid Residue (%) of filaments from TGA curves.

| Sample | $T_{ONSET}$ (°C) | $T_{95\%\ weight\ loss}$(°C) | Solid Residue (%) |
|---|---|---|---|
| PLA | 283.1 | 401.0 | 0.4 ± 0.1 |
| PETG | 351.6 | 584.1 | 2.9 ± 0.3 |

Finally, thermal analysis and mechanical tests were performed on printed PLA and PET samples (labeled PLA 3D and PETG 3D). The overall results are shown in Table S2. As expected, the FFF printing process, and thus the melting of the materials, changes the molecular structure and length of the polymer chains of the materials. Materials are usually subjected to thermo-mechanical degradation during the printing process,

which results in a reduction in molecular weight, in the respective glass transition temperatures and an increase in crystallinity. In this specific case, the DSC analysis performed on the 3D-printed bars does not show any significant changes in the thermal properties, and therefore there were no evident structural changes in the PLA and PETG materials. Mechanical test data show a different behavior of the two materials at different stresses. However, the results are comparable with those reported in the scientific literature [1,2,13,14]. Overall, Table S2 shows a higher strength of PETG than PLA, especially at tensile stress. For all these reasons, PETG was selected as the most suitable material for the realization of the 3D copy of the column.

**Table S2**. Thermal and mechanical properties of PLA 3D and PETG 3D bars.

| Sample | Thermal properties | | Tensile properties | | | Flexural properties | | |
|---|---|---|---|---|---|---|---|---|
| | $T_g$(°C) | $T_m$ (°C) | E (GPa) | ε (%) | σ (MPa) | E (GPa) | ε (%) | σ (MPa) |
| PLA 3D | 56.5 | 166.1 | 1.1 ± 0.2 | 3.6 ± 0.4 | 38.2 ± 5.1 | 2.9 ± 0.1 | 3.3 ± 0.3 | 66.9 ± 4.1 |
| PETG 3D | 70.8 | 254.0 | 1.7 ± 0.3 | 5.2 ± 0.9 | 40.9 ± 6.2 | 1.4 ± 0.1 | 3.6 ± 0.5 | 67.1 ± 1.9 |

**References**


1. Fico, D.; Rizzo, D.; Casciaro, R.; Esposito Corcione, C. A Review of Polymer-Based Materials for Fused Filament Fabrication (FFF): Focus on Sustainability and Recycled Materials. *Polymers (Basel).* **2022**, *14*, 465, doi:https://doi.org/10.3390/ polym14030465.

2. Fico, D.; Rizzo, D.; De Carolis, V.; Montagna, F.; Esposito Corcione, C. Sustainable Polymer Composites Manufacturing through 3D Printing Technologies by Using Recycled Polymer and Filler. *Polymers (Basel).* **2022**, *14*, 3756, doi:https://doi.org/ 10.3390/polym14183756.

3. Cano-Vicent, A.; Tambuwala, M.M.; Hassan, S.S.; Barh, D.; Aljabali, A.A.A.; Birkett, M.; Arjunan, A.; Serrano-Aroca, Á. Fused Deposition Modelling: Current Status, Methodology, Applications and Future Prospects. *Addit. Manuf.* **2021**, *47*, doi:10.1016/j.addma.2021.102378.

4. Wang, S.; Capoen, L.; D'hooge, D.R.; Cardon, L. Can the Melt Flow Index Be Used to Predict the Success of Fused Deposition Modelling of Commercial Poly(Lactic Acid) Filaments into 3D Printed Materials? *Plast. Rubber Compos.* **2018**, *47*, 9–16, doi:10.1080/14658011.2017.1397308.

5. Anderson, I. Mechanical Properties of Specimens 3D Printed with Virgin and Recycled Polylactic Acid. *3D Print. Addit. Manuf.* **2017**, *4*, 110–115, doi:10.1089/3dp.2016.0054.

6. Ferrari, F.; Esposito Corcione, C.; Montagna, F.; Maffezzoli, A. 3D Printing of Polymer Waste for Improving People's Awareness about Marine Litter. *Polymers (Basel).* **2020**, *12*, 1738.

7. Groeninckx, G.; Berghmans, H.; Overbergh, N.; Smets, G. Crystallization of Poly(Ethylene Terephthalate) Induced by Inorganic Compounds. I. Crystallization Behaviour from the Glassy State in a Low-Temperature Region. *J. Polym. Sci.* **1974**, *12*, 303–316, doi:10.1002/pol.1974.180120207.

8. Colón Quintana, J.L.; Slattery, L.; Pinkham, J.; Keaton, J.; Lopez-Anido, R.A.; Sharp, K. Effects of Fiber Orientation on the Coefficient of Thermal Expansion of Fiber-Filled Polymer Systems in Large Format Polymer Extrusion-Based Additive Manufacturing. *Materials (Basel).* **2022**, *15*, 1–27, doi:10.3390/ma15082764.

9. Shi, Q.; Xiao, R.; Yang, H.; Lei, D. Effects of Physical Aging on Thermomechanical Behaviors of Poly(Ethylene Terephthalate)-Glycol (PETG). *Polym. Technol. Mater.* **2020**, *59*, 835–846, doi:10.1080/25740881.2019.1695273.

10. Marković, M.P.; Cingesar, I.K.; Keran, L.; Prlić, D.; Grčić, I.; Vrsaljko, D. Thermal and Mechanical Characterization of the New Functional Composites Used for 3D Printing of Static Mixers. *Materials*

*(Basel).* **2022**, *15*, doi:10.3390/ma15196713.

11. Carrasco, F.; Pagès, P.; Gámez-Pérez, J.; Santana, O.O.; Maspoch, M.L. Processing of Poly(Lactic Acid): Characterization of Chemical Structure, Thermal Stability and Mechanical Properties. *Polym. Degrad. Stab.* **2010**, *95*, 116–125, doi:10.1016/j.polymdegradstab.2009.11.045.

12. Esposito Corcione, C.; Palumbo, E.; Masciullo, A.; Montagna, F.; Torricelli, M.C. Fused Deposition Modeling (FDM): An Innovative Technique Aimed at Reusing Lecce Stone Waste for Industrial Design and Building Applications. *Constr. Build. Mater.* **2018**, *158*, 276–284, doi:10.1016/j.conbuildmat.2017.10.011.

13. Fico, D.; Rizzo, D.; De Carolis, V.; Montagna, F.; Palumbo, E.; Corcione, C.E. Development and Characterization of Sustainable PLA/Olive Wood Waste Composites for Rehabilitation Applications Using Fused Filament Fabrication (FFF). *J. Build. Eng.* **2022**, *56*, 104673, doi:10.1016/j.jobe.2022.104673.

14. Valvez, S.; Silva, A.P.; Reis, P.N.B. Optimization of Printing Parameters to Maximize the Mechanical Properties of 3D-Printed PETG-Based Parts. *Polymers (Basel).* **2022**, *14*, doi:10.3390/polym14132564.
